# Combination of clinical symptoms and blood biomarkers can improve discrimination between bacterial or viral community-acquired pneumonia in children

**DOI:** 10.1186/s12890-019-0835-5

**Published:** 2019-04-02

**Authors:** Mejbah U. Bhuiyan, Christopher C. Blyth, Rachel West, Jurissa Lang, Tasmina Rahman, Caitlyn Granland, Camilla de Gier, Meredith L. Borland, Ruth B. Thornton, Lea-Ann S. Kirkham, Andrew Martin, Peter C. Richmond, David W. Smith, Adam Jaffe, Thomas L. Snelling

**Affiliations:** 1 Division of Paediatrics, School of Medicine, Faculty of Health and Medical Sciences, The University of Western Australia, Perth, Australia; 2 Wesfarmers Centre of Vaccines and Infectious Diseases, Telethon Kids Institute, Perth, Australia; 3 Perth Children’s Hospital, Perth, Australia; 4 Department of Microbiology, PathWest Laboratory Medicine WA, Perth, Australia; 5 School of Biomedical Sciences, Faculty of Health and Medical Sciences, The University of Western Australia, Perth, Australia; 6 Division of Emergency Medicine, School of Medicine, Faculty of Health and Medical Sciences, The University of Western Australia, Perth, Australia; 7 School of Women’s and Children’s Health, Faculty of Medicine, University of New South Wales, Sydney, Australia

**Keywords:** Blood biomarker, C-reactive protein, Children, Pneumonia, Bacteria, Virus

## Abstract

**Background:**

Differentiating bacterial from viral pneumonia is important for guiding targeted management and judicious use of antibiotics. We assessed if clinical characteristics and blood inflammatory biomarkers could be used to distinguish bacterial from viral pneumonia.

**Methods:**

Western Australian children (≤17 years) hospitalized with radiologically-confirmed community-acquired pneumonia were recruited and clinical symptoms and management data were collected. C-reactive protein (CRP), white cell counts (WCC) and absolute neutrophil counts (ANC) were measured as part of routine care. Clinical characteristics and biomarker levels were compared between cases with definite bacterial pneumonia (clinical empyema and/or bacteria detected in blood or pleural fluid), presumed viral pneumonia (presence of ≥1 virus in nasopharyngeal swab without criteria for definite bacterial pneumonia), and other pneumonia cases (pneumonia in the absence of criteria for either definite bacterial or presumed viral pneumonia). The area-under-curve (AUC) of the receiver operating characteristic (ROC) curve for varying biomarker levels were used to characterise their utility for discriminating definite bacterial from presumed viral pneumonia. For biomarkers with AUC > 0.8 (fair discriminator), Youden index was measured to determine the optimal cut-off threshold, and sensitivity, specificity, predictive values (positive and negative) were calculated. We investigated whether better discrimination could be achieved by combining biomarker values with the presence/absence of symptoms.

**Results:**

From May 2015 to October 2017, 230 pneumonia cases were enrolled: 30 with definite bacterial pneumonia, 118 with presumed viral pneumonia and 82 other pneumonia cases. Differences in clinical signs and symptoms across the groups were noted; more definite bacterial pneumonia cases required intravenous fluid and oxygen supplementation than presumed viral or other pneumonia cases. CRP, WCC and ANC were substantially higher in definite bacterial cases. For a CRP threshold of 72 mg/L, the AUC of ROC was 0.82 for discriminating definite bacterial pneumonia from presumed viral pneumonia. Combining the CRP with either the presence of fever (≥38^ο^C) or the absence of rhinorrhea improved the discrimination.

**Conclusions:**

Combining elevated CRP with the presence or absence of clinical signs/ symptoms differentiates definite bacterial from presumed viral pneumonia better than CRP alone. Further studies are required to explore combination of biomarkers and symptoms for use as definitive diagnostic tool.

**Electronic supplementary material:**

The online version of this article (10.1186/s12890-019-0835-5) contains supplementary material, which is available to authorized users.

## Background

Globally, pneumonia is the leading cause of hospitalizations and death among children with nearly 120 million new cases and one million deaths each year [1]. In Australia, pneumonia is associated with 5–8 hospitalisations per 1000 child-years among children < 5 years old, with deaths being rare [2]. Australian Aboriginal children are 14 times more at risk of infectious diseases than non-Aboriginal children [3].

Respiratory bacteria and viruses are frequently detected in specimens collected from children with pneumonia [4]. Identifying the infectious agents associated with illness can guide management of the infection and facilitate judicious use of antibiotics. Differentiating bacterial from viral pneumonia based on clinical characteristics is challenging as the clinical signs and symptoms overlap [5, 6].

Despite the growing availability of molecular techniques for pathogen detection, including quantitative and qualitative pathogen detection, laboratory results are usually only available after treatment decisions have been made. Several studies have assessed the utility of non-specific inflammatory biomarkers such as C-reactive protein (CRP), an acute-phase reactant released in response to cytokine interleukin-6, white cell count (WCC) and absolute neutrophil count (ANC) to discriminate probable bacterial infections from non-bacterial infections and also to assess the severity of illness [7, 8]. Bacterial pneumonia has been associated with higher CRP, WCC and ANC than viral pneumonia [8–11], while some studies have found no difference in biomarkers among bacterial and viral cases of pneumonia [12, 13]. Even studies that report differences in these biomarkers cannot determine any reliable thresholds for differentiating bacterial pneumonia from viral pneumonia [10, 14]. Previous studies were also affected by small sample size, use of less sensitive methods for pathogen detection which could have resulted an inaccurate categorization of bacterial and viral cases [12, 14]. Furthermore, studies have been conducted in low-income settings where the contribution of bacterial infection may be higher and where children are at higher risk of other infectious diseases that could influence the biomarker levels and confound the analysis [10].

The aetiology of childhood pneumonia has changed in high-income settings with routine pneumococcal conjugate vaccination program – decrease in bacterial aetiology and increase in viral aetiology have been reported [15, 16]. However, the utility of inflammatory biomarkers in a highly-vaccinated paediatric population with pneumonia to differentiate bacterial from viral pneumonia has rarely been assessed [11]. We therefore assessed the distribution of inflammatory biomarkers (CRP, WCC and ANC) in blood samples from a prospective case-control study of Western Australian children with radiologically-confirmed pneumonia [17]. We compared the clinical characteristics and biomarkers level among pneumonia cases detected with bacteria and viruses. We assessed the cut-off threshold of these biomarkers for discriminating bacterial pneumonia from viral pneumonia. Findings from this study could help in developing a rapid point-of-care diagnostic tool or algorithm to predict the likely causative pathogen and to assist clinicians to target management of childhood pneumonia.

## Methods

### Study population

From May 15, 2015 through October 31, 2017, we prospectively enrolled children aged ≤17 years with radiologically-confirmed community-acquired pneumonia (further mentioned as case), hospitalized at the Princess Margaret Hospital for Children (PMH), Perth, Australia (now known as Perth Children’s Hospital). The study hospital is the only publically-funded tertiary paediatric hospital for a total population of 2.6 million in Western Australia. We followed a pragmatic definition of radiologically-confirmed pneumonia (infiltrates or alveolar consolidation as determined by the treating clinician) facilitating recruitment after hours (i.e. when a radiologist is not available to review the chest x-ray) [18]. The study design and eligibility criteria were published previously [17]. Existing or ever diagnosed with any comorbidity was not considered as exclusion criteria.

### Demographic and clinical data collection

A structured questionnaire was administered to parents/guardians to record demographic and clinical information including symptoms. Clinical observations including respiratory rate and oxygen saturation at presentation, highest measured temperature, and need for intravenous fluid, oxygen supplementation, and respiratory support were recorded from review of the medical notes.

### Specimen collection and laboratory procedures

As part of medical care, a blood specimen was collected from each case by the treating clinician and tested for C-reactive protein (CRP) concentration, white cell count (WCC), absolute neutrophil count (ANC) count and blood culture at the hospital laboratory. Pleural fluid was drained from case with pleural effusion and assessed by microscopy, culture and polymerase chain reaction (PCR) for bacterial pathogens. A nasopharyngeal swab (NPS; FLOQSwabs; Copan Diagnostics, Murrieta, CA) was collected following standard sample collection procedure and within 36 h of presentation of hospital presentation [19]. The swab was tested for a total of 14 respiratory viruses: influenza A/H1N1, A/H3N2 and B, respiratory syncytial virus (RSV), human parainfluenza virus (HPIV) type 1–3, human metapneumovirus (HMPV), adenovirus (AV), rhinovirus (RV), human coronavirus (HCoV OC43, HCoV 229E, HCoV HKU1 and HCoV NL63) and 6 bacteria: *Streptococcus pneumoniae*, *Staphylococcus aureus, Moraxella catarrhalis*, *Haemophilus influenzae, Mycoplasma pneumoniae* and *Chlamydophila pneumoniae* using polymerase chain reaction (PCR). Laboratory procedures including nucleic acid extraction and the primers and probes used in the PCRs have been described previously [17].

### Data management and analysis

Age-specific tachypnoea was defined per World Health Organization criteria as: respiratory rate of ≥60 breaths/min in children aged < 2 months, ≥50 breaths/min in children aged 2–12 months and ≥ 40 breaths/min in children aged > 1 year [20]. Disease severity for each case was assessed by Respiratory Index of Severity in Children (RISC) score using severity of respiratory signs on physical examination during hospital presentation and following WHO child growth standard [21]. The RISC score could range from − 2 to 6 with higher scores indicating increased severity. The cases were categorised into three distinct groups based on probable pneumonia aetiology: (a) definite bacterial cases: cases with clinical empyema or with at least one putative bacterial pathogen detected in blood or pleural fluid by culture or PCR (regardless of the detection of respiratory viruses in NPS); (b) presumed viral cases: cases without empyema or bacteria detected in blood/ pleural fluid, and with at least one respiratory virus detected in NPS (with or without bacteria detected in the NPS); (c) other pneumonia cases: cases fulfilling neither of the criteria for definite bacterial or presumed viral (with or without bacteria detected in NPS).

The frequency of clinical characteristics was reported for each group. For categorical clinical characteristics, Chi-square or Fisher’s exact test, whichever appropriate, was used to compare frequencies between groups. For continuous variables, means (range) were reported if the data were normally distributed, otherwise, medians (interquartile range, IQR) were reported. *Students’ t-test* or Wilcoxon rank-sum test, whichever appropriate, was used to compare between groups.

For CRP, WCC and ANC concentration, the area-under-curve (AUC) for the receiver-operating-characteristic (ROC) curve to describe the ability of each marker to differentiate definite bacterial pneumonia from presumed viral and other pneumonia cases was measured. The diagnostic performance of the biomarker for differentiating bacterial pneumonia was assessed by the value the area-under-curve (AUC): 0.9–1.0 was considered excellent discrimination, 0.8–0.9 as good, 0.7–0.8 as fair, 0.6–0.7 as poor and 0.5–0.6 as non-discriminatory [22]. Sensitivity, specificity, positive predictive value (PPV) and negative predictive value (NPV) at different cut-off values of those biomarkers with AUC value > 0.8 was assessed. Youden index was assessed to identify the optimum cut-off to distinguish definite bacterial from presumed viral pneumonia, and from presumed viral plus other pneumonia cases [23]. We further explored if combination of symptoms and biomarkers improved the overall sensitivity and specificity for discriminating bacterial and viral pneumonia. All statistical analyses were conducted in STATA 13.0 and plots were prepared using GraphPad Prism 5.0.

## Results

A total of 230 children with radiologically confirmed community-acquired pneumonia (cases) were enrolled during the study period. Of these, 120 (52%) were male and 147 (64%) aged ≤ 5 years; the median age was 38 months (IQR: 19, 81). Twenty-one (9%) cases were Aboriginal. Of the 230 cases, 210 (91%) had received at least 2 doses of the 13-valent pneumococcal conjugate vaccine. Demographics and existing or ever diagnosed with co-morbidities of the enrolled children are summarized in Table 1.

**Table 1:** Characteristics of children with community-acquired pneumonia (cases) and healthy children (controls), Perth, Western Australia, May 2015 – October 2017

Parameter	Case (%) (*N* = 230)
Demographic and clinical data	
Age	
●< 12 months	21 (9.1)
●1–5 years	126 (54.7)
●6–9 years	60 (26.1)
●10+ years	23 (10)
●Male sex	120 (52.1)
●Aboriginal	21 (9.1)
●Premature	32 (13.9)
Smoker present in household	38 (16.5)
Existing health conditions	
●Any co-morbidity	34 (14.7)
●Immunodeficiency^a^	7 (3.0)
●immunocompromised condition	5 (2.1)
●congenital abnormality^b^	17 (7.3)
●chronic respiratory illness	9 (3.9)
●chronic neuromuscular disorder illness	9 (3.9)
●Other^c^	1 (0.4)

a: IgG subclass deficiency (n = 1); Low IgA (*n* = 1); T-cell deficiency (*n* = 1), Mannose-binding lectin deficiency (n = 2), DiGeorge’s syndrome (*n* = 2)

b: Capillary malfunction syndrome (n = 1); Beckwith-Wiedemann syndrome and congenital hypothoroidism (n = 2); Atrioventricular septal defect (n = 1); Down syndrome (*n* = 3); Sotos syndrome (n = 1); Spinal muscular atrophy type 2 (n = 1); Developmental delay (n = 1); Congenital heart disease (n = 2); Prader Willi syndrome (n = 1); Congenital sensorineural deafness (n = 1); Ehlers-Danlos syndrome (n = 1); Gasroschisis (n = 1); Cleft lip (n = 1);

c: Intracranial shunt (n = 1)

The median length of hospitalization was 2 days (IQR: 1, 3) and all children were discharged with no deaths. At hospital presentation, 38 (17%) cases had blood oxygen saturation (SPO_2_) level ≤ 92% and tachypnoea observed in 88 (38%). Nearly half of cases (109/230) received antibiotics in the 7 days prior to hospital presentation and all but three (227/230, 99%) received antibiotics during hospital stay. Twenty-four cases were diagnosed with pleural effusion and of these, 21 (88%) had pleural fluid drained: all 21 had microscopic purulence consistent with empyema.

There were 30 (13%) cases of definite bacterial pneumonia: 9 with bacteraemia, 15 with empyema, and 6 with both bacteraemia and empyema. Of the 21 pleural fluid samples from empyema cases, 1 was culture and PCR positive, and 9 were PCR positive (only) for *Streptococcus pneumoniae*, 1 each cultured methicillin-resistant *Staphylococcus aureus*, methicillin-sensitive *Staphylococcus aureus*, and *Streptococcus pyogenes*; and 1 sample was PCR positive for *Mycoplasma pneumoniae.* At least one respiratory virus was detected in NPS of 12 of the 30 cases with definite bacterial pneumonia. Of the remaining 200 cases, at least one virus was detected in nasopharyngeal swab from 118 (59%) cases including 98 with co-detection of respiratory bacteria. Among these 118 presumed viral pneumonia cases, 43 (36%) had RSV detected, 31 (26%) had rhinovirus, 21 (18%) had HMPV, 15 (13%) had influenza and 9 (8%) each had adenovirus and parainfluenza. No virus was detected in the nasopharyngeal swabs of 82 (41%) cases (other pneumonia) including 51 had detectable respiratory bacteria on NPS. The distribution of bacteria detected in NPS in three case groups of pneumonia are presented in Additional file 1: Table S1. The distribution of bacteria in NPS in three case groups were similar. Among cases in three groups, 53% of definite bacterial pneumonia cases, 40% of presumed viral pneumonia cases and 56% of other pneumonia cases received antibiotics before hospitalization.

The clinical features of cases and medical interventions are summarized in Table 2. Few differences in clinical symptoms and signs were observed across the different groups (Table 2). Fever (defined as temperature ≥ 38.0 °C) was more frequently observed in definite bacterial pneumonia than in presumed viral (*p* = 0.002) or other pneumonia cases (*p* < 0.001). Rhinorrhoea was more frequent in presumed viral pneumonia than in either definite bacterial pneumonia (*p* < 0.001) or other pneumonia cases (*p* < 0.001). Age-specific tachypnoea was also more common in presumed viral pneumonia than in definite bacterial (*p* = 0.08) or other pneumonia cases (*p* = 0.003). More definite bacterial pneumonia cases required intravenous fluid therapy than presumed viral (*p* = 0.02) or other pneumonia cases (*p* = 0.001). Furthermore, more than half (53%) of definite bacterial cases required both supplemental O_2_ and intravenous fluid, substantially higher than presumed viral (32%; *p* = 0.03) or other pneumonia cases (23%, *p* = 0.002) (Table 2). Definite bacterial pneumonia cases also had a greater median length of hospital stay (6.5 days, 9 days with empyema and 2 days without) than presumed viral (2 days) or other pneumonia (2 days) (*p* < 0.001 for each). The mean RISC severity score was 1.2 (range: 0, 5) for definite bacterial cases, compared to 1.0 (− 2, 5) for presumed viral and 0.8 (− 2, 4) for other pneumonia cases, respectively.

**Table 2:** Distribution of clinical characteristics, management and concentration of inflammatory biomarkers in children with definite bacterial pneumonia, presumed viral pneumonia and other pneumonia

Parameter	Definite bacterial pneumonia (*N* = 30), A	Presumed viral pneumonia, (*N* = 118) B	Other pneumonia, (*N* = 82) C
Clinical features			
Fever (body temperature ≥ 38.0 °C)	27 (90) λ** μ***	71 (60)	41 (50)
Age-specific Tachypnoea	9 (30)	56 (47) δ**	22 (27)
SpO_2_% at presentation, median (IQR)	96 (94, 98)	95 (93, 98)	95 (94, 97)
Diagnosis of wheeze at presentation	0 (0)	18 (15)	11 (13)
Diagnosis of crackles/crepitation at presentation	8 (27)	58 (49)	42 (52)
Cough	24 (80)	109 (92)	75 (91)
Rhinorrhea	12 (40)	93 (79) δ*** λ ***	41 (50)
Difficulty in breathing	25 (83)	88 (75)	62 (76)
Vomiting	19 (63)	78 (66)	39 (48)
Body rash	3 (10)	14 (12)	12 (15)
Diarrhea	8 (27)	34 (29)	15 (18)
Poor oral intake	22 (73)	82 (69)	61 (74)
Clinical management			
Supplemental O_2_	16 (53)	65 (55)	35 (43)
Intravenous fluid	22 (73) μ*** λ*	60 (51) δ**	25 (31)
Respiratory support	1 (3)	2 (2)	3 (4)
Supplemental O_2 +_ intravenous fluid	16 (53) μ** λ*	38 (32)	19 (23)
Hospitalization day, median (IQR)	8 (4, 11) μ*** λ***	2 (1, 3)	2 (1, 3)
Blood inflammatory markers			
WCC count (×10^9^/L), median (IQR)	16 (11, 21) λ*	11 (8, 18)	12 (8, 18)
CRP (mg/L), median (IQR)	174 (64, 246) λ*** μ***	24 (13, 56)	27 (17, 59)
Absolute neutrophil, (×10^9^/L), median (IQR)	13 (8, 19) λ** μ**	7 (4, 13)	8 (4, 13)

Data are frequency (percentage), unless otherwise mentioned

For comparison, Chi-square test for categorical variables and Wilcoxon-ranksum test for continuous variable was done; *p < 0.05; **p < 0.01; ***p < 0.001

λ Comparison between A and B

μ Comparison between A and C

δ Comparison between B and C

The median blood CRP concentration was more than 6 times higher in definite bacterial cases than in presumed viral (174 versus 24 mg/L; p < 0.001) and other pneumonia cases (174 versus 27 mg/L; p < 0.001). The CRP, WCC and ANC did not vary significantly between presumed viral and other pneumonia cases and between empyema and bacteraemia cases (Table 2, Additional file 1: Table S2). The blood biomarker values did not vary significantly between presumed viral cases with different viral pathogens detected (data not shown).

**Fig. 1:**
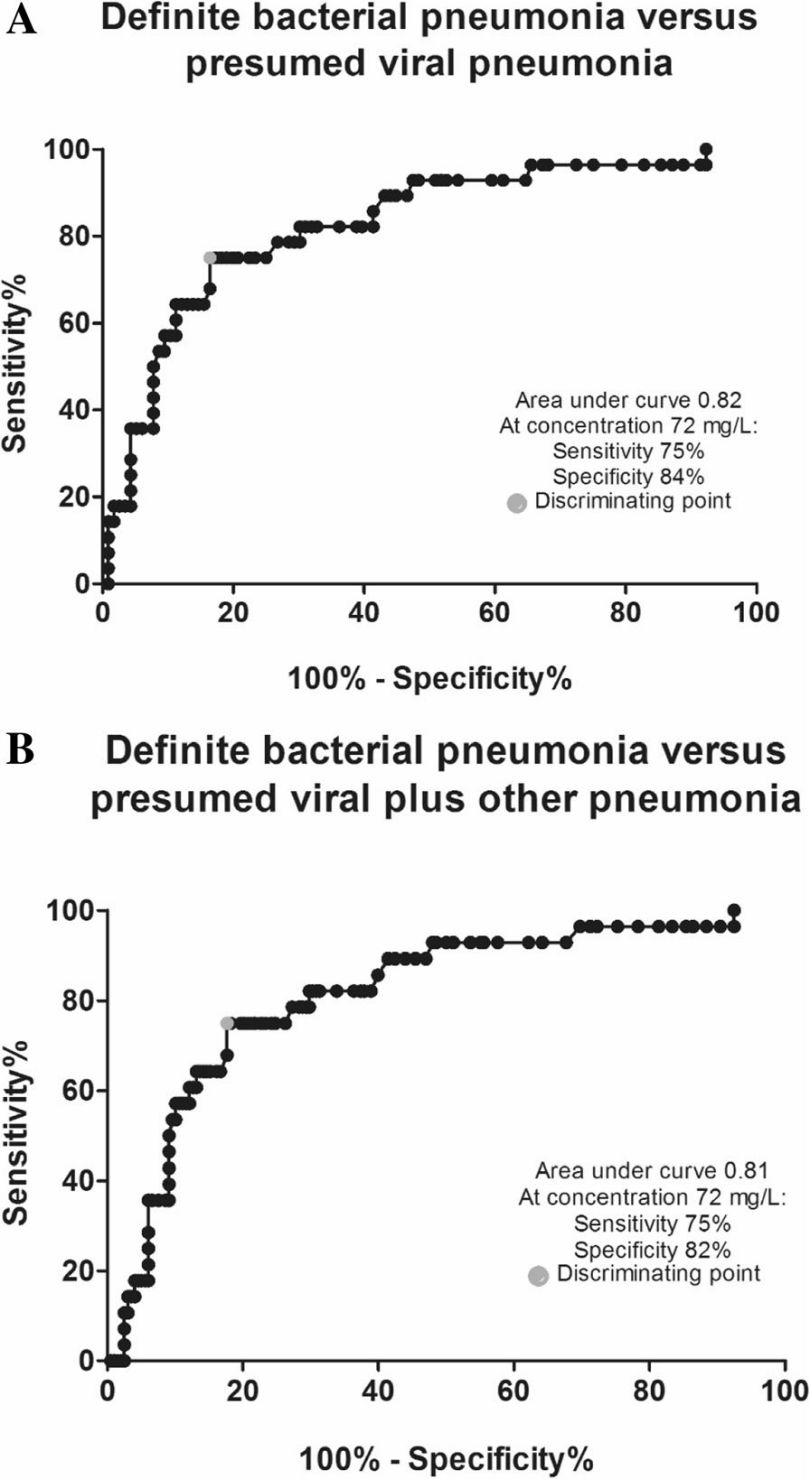
**a**: ROC curve for CRP for differentiating definite bacterial pneumonia against presumed viral pneumonia in radiologically confirmed CAP cases. **b**: ROC curve for CRP for differentiating definite bacterial pneumonia against presumed viral plus other pneumonias in radiologically confirmed CAP cases

The AUC for CRP was 0.82 (95% CI: 0.73, 0.91) for discriminating definite bacterial from presumed viral pneumonia (Fig. 1a) and 0.81 (95% CI: 0.72, 0.89) for discriminating definite bacterial from presumed viral plus other pneumonias (Fig. 1b). For WCC, the AUCs were 0.63 (95% CI: 0.53, 0.74) and 0.65 (95% CI: 0.53, 0.76) for discriminating definite bacterial from presumed viral plus others pneumonias, and definite bacterial from presumed viral pneumonia, respectively (Additional file 2: Figure S1a, 1b). For ANC, the AUCs were 0.68 (95% CI: 0.58, 0.78) and 0.69 (95% CI: 0.58–0.79) for discriminating definite bacterial from presumed viral plus other pneumonias, and definite bacterial from presumed viral pneumonias, respectively (Additional file 2: Figure S2a, 2b). Based on these AUCs, we further assessed the sensitivity, specificity, PPV and NPV at CRP cut-offs of ≥ 40 mg/L, ≥ 60 mg/L and ≥ 100 mg/L, for differentiating bacterial from presumed viral plus other pneumonias, and definite bacterial from presumed viral pneumonias (Additional file 1: Table S3). CRP ≥ 40 mg/L, CRP ≥ 60 mg/L and CRP ≥ 100 mg/L cut-off had sensitivity of 83, 75 and 67%, respectively for differentiating definite bacterial pneumonia from presumed viral pneumonias. From the Youden index, the optimal CRP threshold of ≥72 mg/L was found to discriminate definite bacterial from presumed viral plus other pneumonias with sensitivity 75% (95% CI: 55, 89), specificity 82% (95% CI: 76, 87), PPV 38% and NPV 96%; for discriminating definite bacterial from presumed viral pneumonias the sensitivity was 75% (95% CI: 55, 89), specificity 84% (95% CI: 76, 90), PPV 53% and NPV 93% (Table 3).

**Table 3:** Sensitivity, specificity, positive predictive value (PPV) and negative predictive value (NPV) at CRP threshold value and in combination with clinical symptoms to differentiate definite bacterial pneumonia from presumed viral and other pneumonias

	Definite bacterial versus presumed viral pneumonia				Definite bacterial versus presumed viral plus other pneumonias			
CRP cut-off level (mg/L)	Sensitivity	Specificity	PPV	NPV	Sensitivity	Specificity	PPV	NPV
≥ 72 alone	75	84	53	93	75	82	38	96
≥ 72 and fever	73	90	63	93	73	89	48	96
≥ 72 and absence of rhinorrhoea	65	98	87	93	65	93	59	96
≥ 72 and absence of tachypnoea	65	90	54	93	65	89	38	96

Significant differences in symptoms and signs were then included to assess the impact on the diagnostic performance of the algorithm (Table 3; Additional file 1: Table S3)**.** The combination of CRP (≥72 mg/L) with either the presence of fever or absence of rhinorrhea, improved the specificity and PPV for differentiating bacterial pneumonia from presumed viral plus other pneumonias compared to the CRP alone, with little loss of sensitivity and NPV. While 73% of cases with definite bacterial pneumonia had both fever and elevated CRP (≥72 mg/L), only 11% of presumed viral plus other pneumonias and 10% of presumed viral pneumonias did. Similarly, 65% of definite bacterial pneumonia had elevated CRP (≥72 mg/L) and the absence of rhinorrhea, compared to 7% of presumed viral and other pneumonias, and 2% of presumed viral pneumonias. The PPV for the combination (CRP ≥72 mg/L + fever) and (CRP ≥72 mg/L + absence of rhinorrhea) was 48 and 59% for discriminating definite bacterial from presumed viral plus other pneumonia, and was 63 and 87%, respectively, for discriminating definite bacterial from presumed viral pneumonia, respectively (Table 3).

## Discussion

This study describes the clinical characteristics of children with radiologically confirmed pneumonia, and assesses the utility of serum biomarkers and clinical signs and symptoms for differentiating definite bacterial from other pneumonias in a highly vaccinated population. There were few differences between children with definite bacterial pneumonia, and those with presumed viral pneumonia, and other pneumonias which were neither definite bacterial nor presumed viral. CRP, WCC and ANC were higher in definite bacterial pneumonia and the CRP had value for distinguishing these from presumed viral and other pneumonias. The combination of high CRP with either fever ≥38.0 °C or with absence of rhinorrhoea increased the specificity and PPV compared to elevated CRP alone with little loss in sensitivity, suggesting that combining biomarkers with clinical features is of diagnostic value.

Timely identification of pneumonia aetiology could improve clinical management including decisions about the use of antibiotics. In line with previous studies, the clinical signs and symptoms among cases of definite bacterial and presumed viral pneumonia overlapped and were insufficiently specific in themselves to reliably differentiate one from the other [5, 24]. Our findings are mostly consistent with previous studies which have associated viral pneumonia with low grade fever, tachypnoea, rhinorrhoea and wheezing [4, 25–27]. We did not attempt to distinguish cases based on specific radiographic features, but others have not found any radiographic feature that can reliably distinguish bacterial from viral pneumonia [14, 28, 29].

There are conflicting reports on the utility of serum inflammatory biomarkers such as CRP, WCC, and ANC for differentiating bacterial from viral pneumonia in children [9–12, 14]. In UK children, Elemraid et al. found a higher CRP, WCC and ANC in those with bacterial compared to viral pneumonia, with a CRP level > 80 mg/L reportedly distinguishing severe bacterial pneumonia from viral pneumonia [9]. The sensitivity and specificity for CRP > 80 mg/L for differentiating bacterial versus viral/atypical pneumonia in children with community-acquired pneumonia was 71 and 52%, respectively, in Norway [11] and was 52 and 72%, respectively, in Finland [14]. We found that CRP had better discriminatory utility (AUC > 0.8) than the WCC or ANC, consistent with findings similar settings [9, 11, 14]. The optimum CRP threshold for differentiating definite bacterial from presumed viral and other pneumonia had only moderate predictive value which could limit its use as a sole criterion for antibiotic decision-making in clinical settings. Serum procalcitonin is not routinely used in our study setting, so we could not assess its value for distinguishing bacterial pneumonia from presumed viral pneumonias. Serum procalcitonin (PCT) has been described as a better biomarker for bacterial sepsis than CRP [30], while other found serum CRP as better predictor for lobar consolidation and pleural effusion than PCT [31].

Other studies have also attempted to discriminate bacterial from viral pneumonia cases using either blood biomarkers [8–10, 12, 13, 32], clinical characteristics [5, 24] or radiological differences [14, 28] with modest success. To our knowledge, however, combining inflammatory biomarkers with clinical symptoms has rarely been explored. We found that compared to high CRP (≥72 mg/L) alone, high CRP and either fever or absence of rhinorrhoea improved the specificity and PPV with little loss in sensitivity, thereby improving the diagnostic accuracy.

Respiratory bacteria are often detected in the nasopharynx of healthy asymptomatic children, but are rarely detected in normally sterile sites like blood and pleural fluid. Furthermore, purulent pleural effusions are generally considered to be of bacterial origin even if bacteria cannot be detected. Respiratory viruses are usually detectable in the nasopharynx of children with viral pneumonias, but may also be detectable in healthy children [16, 33, 34]. In the absence of any gold standard microbiological assays for distinguishing bacterial from viral and mixed infections, we categorised cases based on whether they were highly likely to be bacterial (definite), most likely viral (presumed), or possibly either based on whether bacteria were detected in sterile sites, respiratory viruses were detected in the nasopharynx, or neither. This pragmatic approach to classification reflects the limitations of current methods, and some misclassification is likely to have occurred. Previous studies categorised cases as either bacterial or viral, are were more limited due to lower pathogen detection rates and by the use of less sensitive assays [8, 9, 12, 14, 26]. The strength of this study is that the range of pathogens tested covered virtually all important bacteria and viruses associated with childhood pneumonia using sensitive molecular techniques.

Our study had some limitations. While there were clear differences between the definite bacterial and the presumed viral and other pneumonia cases, it is likely that a number of the latter groups also had bacterial pneumonia or mixed infections and we note that many had detectable respiratory bacteria in their nasopharyngeal swabs (but not in their blood or pleural fluid). Of note, there were differences between presumed viral pneumonia and other pneumonia cases; the latter were less likely to have rhinorrhoea and tachypnoea suggesting they may comprise a higher proportion of bacterial infections than the presumed viral group. Half of all cases had prior exposure to antibiotics at enrolment which might have impacted on the natural progression of signs and symptoms and biomarkers, and the sensitivity of bacterial culture. Sputum samples are often used for diagnosis of community-acquired pneumonia in adults but collection of sputum from children is challenging and studies found limited utility of sputum as diagnostic tool for pneumonia [35].

## Conclusions

Empiric use of antibiotics remains as cornerstone of treating pneumonia in the absence of effective point-of-care diagnostics for differentiating bacterial from viral infection. Many children who have viral pneumonia will continue to receive antibiotics without benefit. Early reliable detection of viral pneumonia, or early exclusion of bacterial pneumonia, could reduce unnecessary antibiotic therapy, thereby mitigating the risk of emerging antibiotic resistance [36]. While we have been unable to identify a single biomarker or clinical feature that could be used to confidently distinguish bacterial from viral pneumonia, our findings suggest there may be utility in more sophisticated algorithms that integrate a number of clinical, microbiological, inflammatory biomarker, or radiological factors to improve pneumonia diagnostics and better targeting therapies.

## Additional files

Additional file 1: **Table S1** shows the distribution of four common respiratory bacteria detected by PCR in the nasopharyngeal swab from children who had definite bacterial pneumonia or presumed viral pneumonia or other pneumonia. This table informs readers if the bacteria in upper respiratory tract, nasopharynx, varies in pneumonia cases who had detectable bacteria in sterile body fluids such as blood and/or pleural fluid compared to those who did not have detectable bacteria in sterile body fluids but had detectable respiratory viruses in nasopharynx and to those who did not have either. **Table S2** shows the distribution three blood biomarkers, CRP, WCC and ANC, among definite bacterial pneumonia cases who had empyema, bacteraemia and both empyema and bacteraemia. We found that definite bacterial cases had increased level of blood biomarkers than presumed viral pneumonia or other pneumonia cases (Table 2). We further analysed within the definite bacterial case group to see if these blood biomarkers varies among complicated pneumonia cases (empyema), cases who had bacteraemia (presence of bacteria in blood) and cases who had both empyema and bacteraemia. **Table S3** shows the different diagnostic values such as sensitivity, specificity, positive predictive value (PPV) and negative predictive value (NPV) for different cut-off values of CRP level to differentiate definite bacterial pneumonia from presumed viral and from presumed viral plus other pneumonias. CRP was found to have capacity to distinguish definite bacterial pneumonia from presumed viral and other pneumonia. While we found CRP level ≥ 72 mg/L was the optimal cut-off point (Table 3), we further assessed the diagnostic values at different CRP levels alone and also in presence/absence of other clinical symptoms to understand the discriminatory analysis between definite bacterial pneumonia and viral and other pneumonia. (DOCX 502 kb)

Additional file 2: **Figure S1 A**. ROC curve for blood WCC concentration for differentiating definite bacterial pneumonia against presumed viral pneumonia in radiologically confirmed CAP cases. **Figure S1 B**. ROC curve for blood WCC concentration for differentiating definite bacterial pneumonia against presumed viral plus other pneumonias in radiologically confirmed CAP cases. **Figure S1** shows the output of discriminatory analysis using ROC curve for blood WCC level. The **Figure S1A** shows the ROC curve for blood WCC to distinct between definite bacterial pneumonia and presumed viral pneumonia. The area-under- curve has been inserted in the figure. The **Figure S1B** shows the ROC curve for blood WCC to distinct between definite bacterial pneumonia and presumed viral pneumonia plus other pneumonia. The area-under- curve has been inserted in the figure **Figure S2 A**: ROC curve for blood ANC concentration for differentiating definite bacterial pneumonia against presumed viral pneumonia in radiologically confirmed CAP cases. **Figure S2 B**. ROC curve for blood ANC concentration for differentiating definite bacterial pneumonia against presumed viral plus other pneumonias in radiologically confirmed CAP cases. **Figure S2** shows the output of discriminatory analysis using ROC curve for blood ANC level. The **Figure S1A** shows the ROC curve for blood ANC to distinct between definite bacterial pneumonia and presumed viral pneumonia. The area-under- curve has been inserted in the figure. The **Figure S2B** shows the ROC curve for blood ANC to distinct between definite bacterial pneumonia and presumed viral pneumonia plus other pneumonia. The area-under- curve has been inserted in the figure. (DOCX 19 kb)

## Abbreviations

ANC: Absolute neutrophil count
AUC: Area-under-curve
AV: Adenovirus
CRP: C-reactive protein
HCoV: Human coronavirus
HMPV: Human metapneumovirus
HPIV: Human parainfluenza virus
IQR: Interquartile range
NPS: Nasopharyngeal swab
NPV: Negative predictive value
PCR: Polymerase chain reaction
PCT: Procalcitonin
PMH: Princess Margaret Hospital for Children
PPV: Positive predictive value
RISC: Respiratory Index of Severity in Children
ROC: Receiver-operating-characteristic
RSV: Respiratory syncytial virus
RV: Rhinovirus
WCC: White cell count

## References

[1] Walker CL, Rudan I, Liu L, Nair H, Theodoratou E, Bhutta ZA (2013). Global burden of childhood pneumonia and diarrhoea. Lancet.

[2] Burgner D, Richmond P (2005). The burden of pneumonia in children: an Australian perspective. Paediatr Respir Rev.

[3] Moore H, Burgner D, Carville K, Jacoby P, Richmond P, Lehmann D (2007). Diverging trends for lower respiratory infections in non-aboriginal and aboriginal children. J Paediatr Child Health.

[4] McIntosh K (2002). Community-acquired pneumonia in children. N Engl J Med.

[5] Huijskens EG, Koopmans M, Palmen FM, van Erkel AJ, Mulder PG, Rossen JW (2014). The value of signs and symptoms in differentiating between bacterial, viral and mixed aetiology in patients with community-acquired pneumonia. J Med Microbiol.

[6] Isaacs D (1989). Problems in determining the etiology of community-acquired childhood pneumonia. Pediatr Infect Dis J.

[7] Flood RG, Badik J, Aronoff SC (2008). The utility of serum C-reactive protein in differentiating bacterial from nonbacterial pneumonia in children: a meta-analysis of 1230 children. Pediatr Infect Dis J.

[8] Korppi M, Heiskanen-Kosma T, Leinonen M (1997). White blood cells, C-reactive protein and erythrocyte sedimentation rate in pneumococcal pneumonia in children. Eur Respir J.

[9] Elemraid MA, Rushton SP, Thomas MF, Spencer DA, Gennery AR, Clark JE (2014). Utility of inflammatory markers in predicting the aetiology of pneumonia in children. Diagn Microbiol Infect Dis.

[10] Higdon MM, Le T, O'Brien KL, Murdoch DR, Prosperi C, Baggett HC (2017). Association of C-Reactive Protein With Bacterial and Respiratory Syncytial Virus-Associated Pneumonia Among Children Aged <5 Years in the PERCH Study. Clin Infect Dis.

[11] Berg AS, Inchley CS, Fjaerli HO, Leegaard TM, Lindbaek M, Nakstad B (2017). Clinical features and inflammatory markers in pediatric pneumonia: a prospective study. Eur J Pediatr.

[12] Heiskanen-Kosma T, Korppi M (2000). Serum C-reactive protein cannot differentiate bacterial and viral aetiology of community-acquired pneumonia in children in primary healthcare settings. Scand J Infect Dis.

[13] Korppi M (2004). Non-specific host response markers in the differentiation between pneumococcal and viral pneumonia: what is the most accurate combination?. Pediatr Int.

[14] Virkki R, Juven T, Rikalainen H, Svedstrom E, Mertsola J, Ruuskanen O (2002). Differentiation of bacterial and viral pneumonia in children. Thorax.

[15] Berg AS, Inchley CS, Aase A, Fjaerli HO, Bull R, Aaberge I (2016). Etiology of pneumonia in a pediatric population with high pneumococcal vaccine coverage: a prospective study. Pediatr Infect Dis J.

[16] Jain S, Williams DJ, Arnold SR, Ampofo K, Bramley AM, Reed C (2015). Community-acquired pneumonia requiring hospitalization among U.S. children. N Engl J Med.

[17] Bhuiyan MU, Snelling TL, West R, Lang J, Rahman T, Borland ML (2018). Role of viral and bacterial pathogens in causing pneumonia among Western Australian children: a case-control study protocol. BMJ Open.

[18] Cherian T, Mulholland EK, Carlin JB, Ostensen H, Amin R, de Campo M (2005). Standardized interpretation of paediatric chest radiographs for the diagnosis of pneumonia in epidemiological studies. Bull World Health Organ.

[19] World Health Organization (WHO) (2003). Manual for the Laboratory Identification and Antimicrobial Susceptibility Testing of Bacterial Pathogens of Public Health Importance in the Developing World.

[20] World Health Organization (WHO) (1995). The mangement of acute respiratory infections in children: practical guidelines for outpatient care.

[21] Reed C, Madhi SA, Klugman KP, Kuwanda L, Ortiz JR, Finelli L (2012). Development of the respiratory index of severity in children (RISC) score among young children with respiratory infections in South Africa. PLoS One.

[22] Carter JV, Pan J, Rai SN, Galandiuk S (2016). ROC-ing along: evaluation and interpretation of receiver operating characteristic curves. Surgery.

[23] Youden WJ (1950). Index for rating diagnostic tests. Cancer.

[24] Korppi M, Don M, Valent F, Canciani M (2008). The value of clinical features in differentiating between viral, pneumococcal and atypical bacterial pneumonia in children. Acta Paediatr.

[25] Ruuskanen O, Lahti E, Jennings LC, Murdoch DR (2011). Viral pneumonia. Lancet..

[26] Juven T, Ruuskanen O, Mertsola J (2003). Symptoms and signs of community-acquired pneumonia in children. Scand J Prim Health Care.

[27] Wei L, Liu W, Zhang XA, Liu EM, Wo Y, Cowling BJ (2015). Detection of viral and bacterial pathogens in hospitalized children with acute respiratory illnesses, Chongqing, 2009-2013. Medicine (Baltimore).

[28] Korppi M, Kiekara O, Heiskanen-Kosma T, Soimakallio S (1993). Comparison of radiological findings and microbial aetiology of childhood pneumonia. Acta Paediatr.

[29] O'Grady KA, Torzilo PJ, Frawley K, Chang AB (2014). The radiological diagnosis of pneumonia in children. Pneumonia.

[30] Simon L, Gauvin F, Amre DK, Saint-Louis P, Lacroix J (2004). Serum procalcitonin and C-reactive protein levels as markers of bacterial infection: a systematic review and meta-analysis. Clinical infectious diseases : an official publication of the Infectious Diseases Society of America.

[31] Agnello L, Bellia C, Di Gangi M, Lo Sasso B, Calvaruso L, Bivona G (2016). Utility of serum procalcitonin and C-reactive protein in severity assessment of community-acquired pneumonia in children. Clin Biochem.

[32] Fan RR, Howard LM, Griffin MR, Edwards KM, Zhu Y, Williams JV (2016). Nasopharyngeal pneumococcal density and evolution of acute respiratory illnesses in young children, Peru, 2009-2011. Emerg Infect Dis.

[33] Rhedin S, Lindstrand A, Hjelmgren A, Ryd-Rinder M, Ohrmalm L, Tolfvenstam T (2015). Respiratory viruses associated with community-acquired pneumonia in children: matched case-control study. Thorax..

[34] Clark JE (2015). Determining the microbiological cause of a chest infection. Arch Dis Child.

[35] Murdoch DR, Morpeth SC, Hammitt LL, Driscoll AJ, Watson NL, Baggett HC (2017). The Diagnostic Utility of Induced Sputum Microscopy and Culture in Childhood Pneumonia. Clin Infect Dis.

[36] World Health Organization (WHO) (2017). Global antimicrobial resistance surveillance system (GLASS) report: early implementation 2016-2017.

