# Supplementary material for: Combination of clinical symptoms and blood biomarkers can improve discrimination between bacterial or viral community-acquired pneumonia in children
Source: BMC Pulm Med. 2019 Apr 2;19:71. doi: 10.1186/s12890-019-0835-5 (PMC6444754; doi:10.1186/s12890-019-0835-5)
Supplement: Supplementary file 1 — Table S1 shows the distribution of four common respiratory bacteria detected by PCR in the nasopharyngeal swab from children who had definite bacterial pneumonia or presumed viral pneumonia or other pneumonia. This table informs readers if the bacteria in upper respiratory tract, nasopharynx, varies in pneumonia cases who had detectable bacteria in sterile body fluids such as blood and/or pleural fluid compared to those who did not have detectable bacteria in sterile body fluids but had detectable respiratory viruses in nasopharynx and to those who did not have either. Table S2 shows the distribution three blood biomarkers, CRP, WCC and ANC, among definite bacterial pneumonia cases who had empyema, bacteraemia and both empyema and bacteraemia. We found that definite bacterial cases had increased level of blood biomarkers than presumed viral pneumonia or other pneumonia cases (Table 2). We further analysed within the definite bacterial case group to see if these blood biomarkers varies among complicated pneumonia cases (empyema), cases who had bacteraemia (presence of bacteria in blood) and cases who had both empyema and bacteraemia. Table S3 shows the different diagnostic values such as sensitivity, specificity, positive predictive value (PPV) and negative predictive value (NPV) for different cut-off values of CRP level to differentiate definite bacterial pneumonia from presumed viral and from presumed viral plus other pneumonias. CRP was found to have capacity to distinguish definite bacterial pneumonia from presumed viral and other pneumonia. While we found CRP level ≥ 72 mg/L was the optimal cut-off point (Table 3), we further assessed the diagnostic values at different CRP levels alone and also in presence/absence of other clinical symptoms to understand the discriminatory analysis between definite bacterial pneumonia and viral and other pneumonia. (DOCX 502 kb) [file 12890_2019_835_MOESM1_ESM.docx]

Table S1: Distribution of respiratory bacteria in nasopharyngeal swab (NPS) from children who had definite bacterial pneumonia or presumed viral pneumonia or other pneumonia

| Type of bacteria detected | **Definite bacterial pneumonia**  **(N=30)** | **Presumed viral pneumonia,**  **(N=118)** | **Other pneumonia, (N=82)** |
| --- | --- | --- | --- |
| *Streptococcus pneumoniae* | 9 (30) | 36 (31) | 14 (17) |
| *Haemophilus influenzae* | 7 (23) | 51 (43) | 17 (21) |
| *Moraxella catarrhalis* | 13 (43) | 68 (58) | 22 (27) |
| *Staphlylococcus aureus* | 5 (17) | 17 (14) | 16 (19) |

Data are frequency (percentage), unless otherwise mentioned

Table S2: Median (IQR) concentration of blood biomarkers CRP, WCC and ANC in cases who had empyema, bacteraemia and both empyema and bacteraemia

| Blood marker | Empyema (n=15) | Bacteraemia (n=9) | Both empyema and bacteraemia (n=6) |
| --- | --- | --- | --- |
| CRP (mg/L) | 175 (116, 189) | 146 (49, 252) | 206 (36, 346) |
| WCC (10^9^/L) | 19 (14, 22) | 10 (8, 19) | 20 (8, 24) |
| ANC (10^9^/L) | 14 (9, 20 ) | 9 (7, 14) | 16 (8, 20) |

Table S3: Sensitivity, specificity, positive predictive value (PPV) and negative predictive value (NPV) for different cut-off values of CRP level to differentiate definite bacterial pneumonia from presumed viral and from presumed viral plus other pneumonias

|  | Definite bacterial versus presumed viral pneumonia | | | | Definite bacterial versus presumed viral plus other pneumonias | | | |
| --- | --- | --- | --- | --- | --- | --- | --- | --- |
| CRP cut-off level (mg/L) | Sensitivity | Specificity | PPV | NPV | Sensitivity | Specificity | PPV | NPV |
| > 40 | 83 | 60 | 35 | 93 | 82 | 62 | 24 | 96 |
| > 40 and fever | 81 | 72 | 44 | 93 | 81 | 74 | 32 | 96 |
| > 40 and absence rhinorrhoea | 75 | 89 | 63 | 93 | 64 | 78 | 20 | 96 |
| > 40 and absence tachypnoea | 75 | 74 | 38 | 93 | 75 | 73 | 25 | 96 |
| > 60 alone | 75 | 77 | 44 | 93 | 75 | 77 | 32 | 96 |
| > 60 and fever | 73 | 83 | 51 | 93 | 73 | 84 | 40 | 96 |
| > 60 and absence rhinorrhoea | 65 | 98 | 72 | 93 | 65 | 92 | 50 | 96 |
| > 60 and absence tachypnoea | 65 | 85 | 45 | 93 | 65 | 64 | 31 | 96 |
| > 100 alone | 67 | 87 | 56 | 92 | 68 | 86 | 40 | 95 |
| > 100 and fever | 65 | 90 | 61 | 92 | 66 | 89 | 47 | 95 |
| > 100 and absence rhinorrhoea | 57 | 98 | 85 | 92 | 57 | 96 | 63 | 95 |
| > 100 and absence tachypnoea | 55 | 93 | 61 | 92 | 55 | 91 | 39 | 95 |
