# Supplementary material for: Combination of clinical symptoms and blood biomarkers can improve discrimination between bacterial or viral community-acquired pneumonia in children
Source: BMC Pulm Med. 2019 Apr 2;19:71. doi: 10.1186/s12890-019-0835-5 (PMC6444754; doi:10.1186/s12890-019-0835-5)

Figure S1 A: ROC curve for blood WCC concentration for differentiating definite bacterial pneumonia against presumed viral pneumonia in radiologically confirmed CAP cases


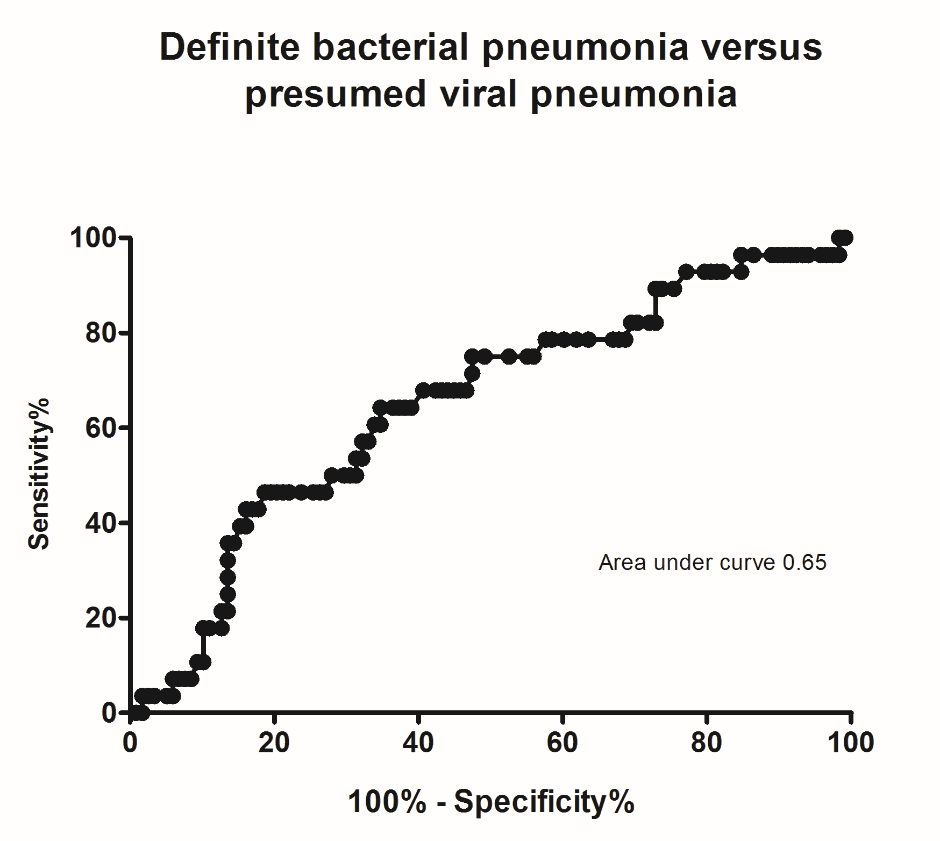


Figure S1 B: ROC curve for blood WCC concentration for differentiating definite bacterial pneumonia against presumed viral plus other pneumonias in radiologically confirmed CAP cases


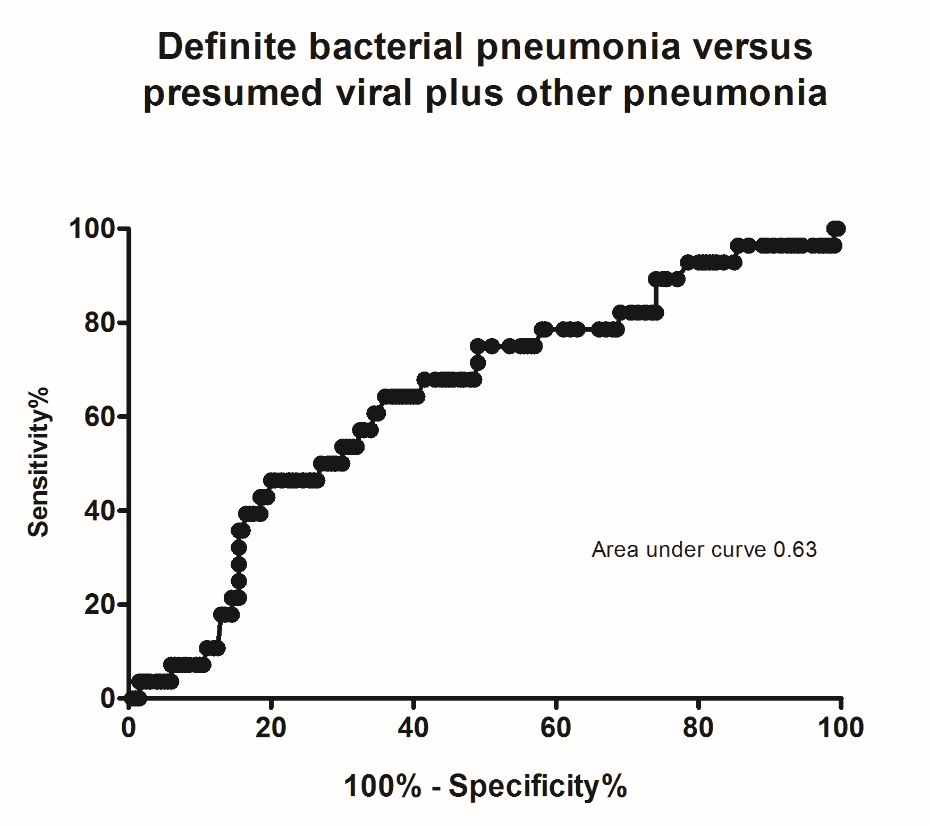


Figure S2 A: ROC curve for blood ANC concentration for differentiating definite bacterial pneumonia against presumed viral pneumonia in radiologically confirmed CAP cases


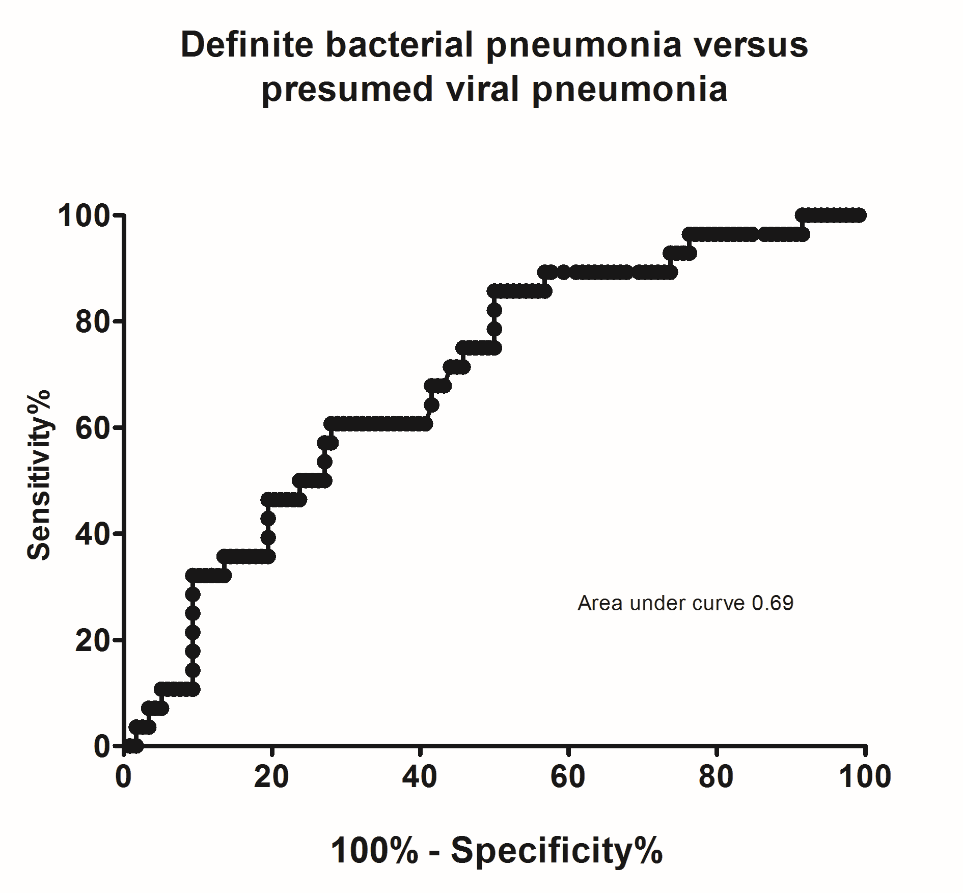


Figure S2 B: ROC curve for blood ANC concentration for differentiating definite bacterial pneumonia against presumed viral plus other pneumonias in radiologically confirmed CAP cases


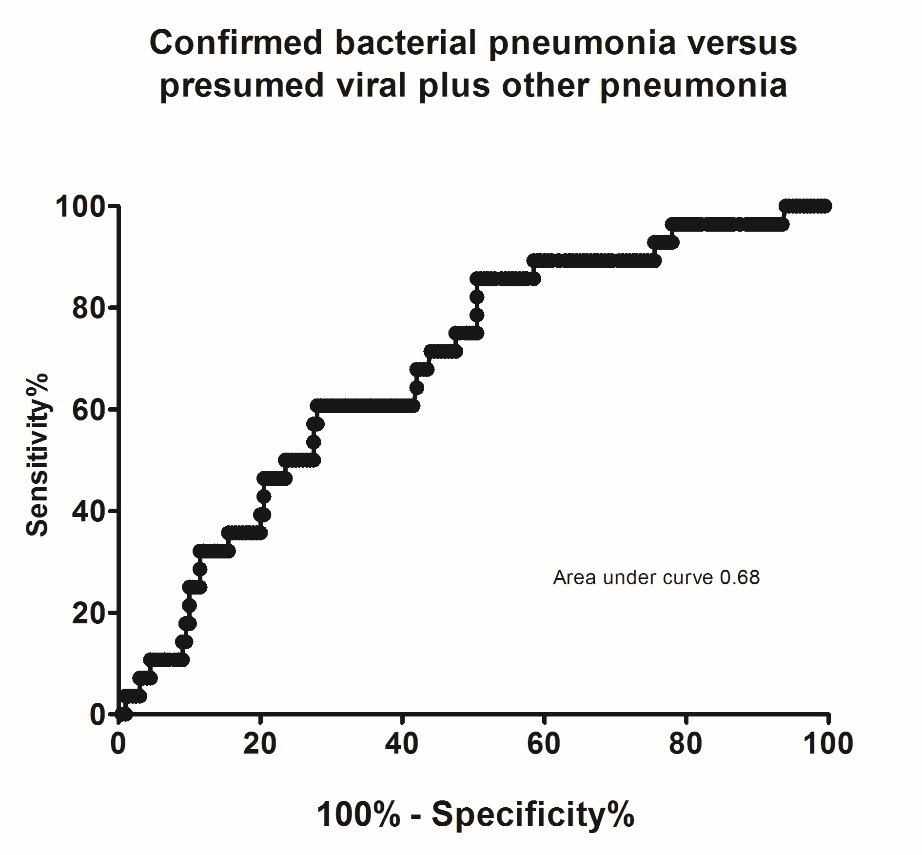

Supplement: Supplementary file 2 — Figure S1 A. ROC curve for blood WCC concentration for differentiating definite bacterial pneumonia against presumed viral pneumonia in radiologically confirmed CAP cases. Figure S1 B. ROC curve for blood WCC concentration for differentiating definite bacterial pneumonia against presumed viral plus other pneumonias in radiologically confirmed CAP cases. Figure S1 shows the output of discriminatory analysis using ROC curve for blood WCC level. The Figure S1A shows the ROC curve for blood WCC to distinct between definite bacterial pneumonia and presumed viral pneumonia. The area-under- curve has been inserted in the figure. The Figure S1B shows the ROC curve for blood WCC to distinct between definite bacterial pneumonia and presumed viral pneumonia plus other pneumonia. The area-under- curve has been inserted in the figure Figure S2 A: ROC curve for blood ANC concentration for differentiating definite bacterial pneumonia against presumed viral pneumonia in radiologically confirmed CAP cases. Figure S2 B. ROC curve for blood ANC concentration for differentiating definite bacterial pneumonia against presumed viral plus other pneumonias in radiologically confirmed CAP cases. Figure S2 shows the output of discriminatory analysis using ROC curve for blood ANC level. The Figure S1A shows the ROC curve for blood ANC to distinct between definite bacterial pneumonia and presumed viral pneumonia. The area-under- curve has been inserted in the figure. The Figure S2B shows the ROC curve for blood ANC to distinct between definite bacterial pneumonia and presumed viral pneumonia plus other pneumonia. The area-under- curve has been inserted in the figure. (DOCX 19 kb) [file 12890_2019_835_MOESM2_ESM.docx]
